# Immune‐related interaction perturbation networks unravel biological peculiars and clinical significance of glioblastoma

**DOI:** 10.1002/imt2.127

**Published:** 2023-07-16

**Authors:** Zaoqu Liu, Yudi Xu, Yuhui Wang, Siyuan Weng, Hui Xu, Yuqing Ren, Chunguang Guo, Long Liu, Zhenyu Zhang, Xinwei Han

**Affiliations:** ^1^ Department of Interventional Radiology The First Affiliated Hospital of Zhengzhou University Zhengzhou China; ^2^ Interventional Institute of Zhengzhou University Zhengzhou China; ^3^ Interventional Treatment and Clinical Research Center of Henan Province Zhengzhou China; ^4^ Department of Neurology The First Affiliated Hospital of Zhengzhou University Zhengzhou China; ^5^ Department of Clinical Laboratory The Third Affiliated Hospital of Zhengzhou University Zhengzhou China; ^6^ Department of Respiratory and Critical Care Medicine The First Affiliated Hospital of Zhengzhou University Zhengzhou China; ^7^ Department of Endovascular Surgery The First Affiliated Hospital of Zhengzhou University Zhengzhou China; ^8^ Department of Hepatobiliary and Pancreatic Surgery The First Affiliated Hospital of Zhengzhou University Zhengzhou China; ^9^ Department of Neurosurgery The First Affiliated Hospital of Zhengzhou University Zhengzhou China

**Keywords:** glioblastoma, immune network, immunotherapy, molecular subtypes

## Abstract

The immune system is an interacting network of plentiful molecules that could better characterize the relationship between immunity and cancer. This study aims to investigate the behavioral patterns of immune‐related interaction perturbation networks in glioblastoma. An immune‐related interaction‐perturbation framework was introduced to characterize four heterogeneous subtypes using RNA‐seq data of TCGA/CGGA glioblastoma tissues and GTEx normal brain tissues. The stability and robustness of the four subtypes were validated in public datasets and our in‐house cohort. In the four subtypes, C1 was an inflammatory subtype with high immune infiltration, low tumor purity, and potential response to immunotherapy; C2, an invasive subtype, was featured with dismal prognosis, telomerase reverse transcriptase promoter mutations, moderate levels of immunity, and stromal constituents, as well as sensitivity to receptor tyrosine kinase signaling inhibitors; C3 was a proliferative subtype with high tumor purity, immune‐desert microenvironment, sensitivity to phosphatidylinositol 3′‐kinase signaling inhibitor and DNA replication inhibitors, and potential resistance to immunotherapy; C4, a synaptogenesis subtype with the best prognosis, exhibited high synaptogenesis‐related gene expression, prevalent isocitrate dehydrogenase mutations, and potential sensitivity to radiotherapy and chemotherapy. Overall, this study provided an attractive platform from the perspective of immune‐related interaction perturbation networks, which might advance the tailored management of glioblastoma.

## Highlights

- An immune‐related interaction‐perturbation framework was introduced, which both considered vital interaction information in the biological network and the relationship between immunity and cancer.
- Four glioblastoma subtypes endowed with distinct clinical outcomes and biological features were identified and validated based on the immune‐related interaction‐perturbation network.
- This taxonomy might be a promising platform to decipher the heterogeneity of glioblastoma and facilitate tailored management.

## Graphical abstract

This study introduced an immune‐related interaction‐perturbation framework, which both considered vital interaction information in the biological network and the relationship between immunity and cancer. Four glioblastoma subtypes endowed with distinct clinical outcomes and biological features were identified from the immune‐related interaction‐perturbation network. The stability and robustness of this taxonomy were validated in public datasets and our in‐house cohort. Our taxonomy might be a promising platform to decipher the heterogeneity of glioblastoma and facilitate tailored management.

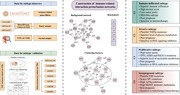

## INTRODUCTION

Glioblastoma (GBM) has been redefined as glioblastoma, isocitrate dehydrogenase (IDH)‐wildtype according to the 2021 WHO classification of central nervous system (CNS) tumors, which is endowed with an infiltrative growth pattern and inherent difficulty in treatment [1, 2]. Surgical resection followed by temozolomide (TMZ) and concomitant radiotherapy is the standard routine for GBM, which remains unsatisfactory outcomes [1, 3, 4]. Tumor heterogeneity may account for the limited efficacy and rapid progression of GBM [5]. Subtype discoveries based on gene expression profiles have been prevalent over the past decade [5, 6]. Recent development in molecular classification has provided critical insights into GBM heterogeneity and facilitated individualized therapies [6, 7]. Nevertheless, bulk RNA‐seq was routinely executed at a specific time or condition, which ignored the fact that biological systems are dynamically altered [7, 8]. Conversely, biological networks containing the information of genes and interactions are relatively stable to time and conditions [9]. A previous study identified four robust subtypes of breast cancer according to the perturbation of gene interaction networks [9]. Moreover, emerging evidence has proven that interaction biomarkers could serve as an effective and reliable tool for distinguishing diseases or phenotypes [10, 11, 12]. Thus, subtype development from the perspective of gene interaction networks might provide new insight into the biological significance and clinical management of GBM.

Immune networks formed by numerous immune molecule and cell interactions play essential roles in various biological processes, especially in tumors [13, 14]. Previous studies have demonstrated that immune networks are inextricably linked to cancer initiation and progression, with the ability to predict prognosis and guide treatment for tumor patients [15, 16]. Hou et al. have demonstrated that the immune‐regulatory interaction networks were tightly associated with unfavorable prognosis of GBM patients [17]. The interplay between tumor cells and the immune microenvironment in GBM ultimately suppresses the beneficial pattern of molecular pathways, improving its malignancy and elevated resistance to cancer therapy [18]. Thus, decoding the tumor heterogeneity based on the interaction perturbation of immune networks is imperative.

From the perspective of immune‐related interaction perturbation networks, this study discovered and validated four heterogeneous subtypes endowed with distinct biological processes and clinical outcomes. This taxonomy might be a promising platform to decipher the heterogeneity of GBM and facilitate tailored management.

## RESULTS

### Subtype discovery from the immune‐related interaction perturbation matrix

**Figure 1:**
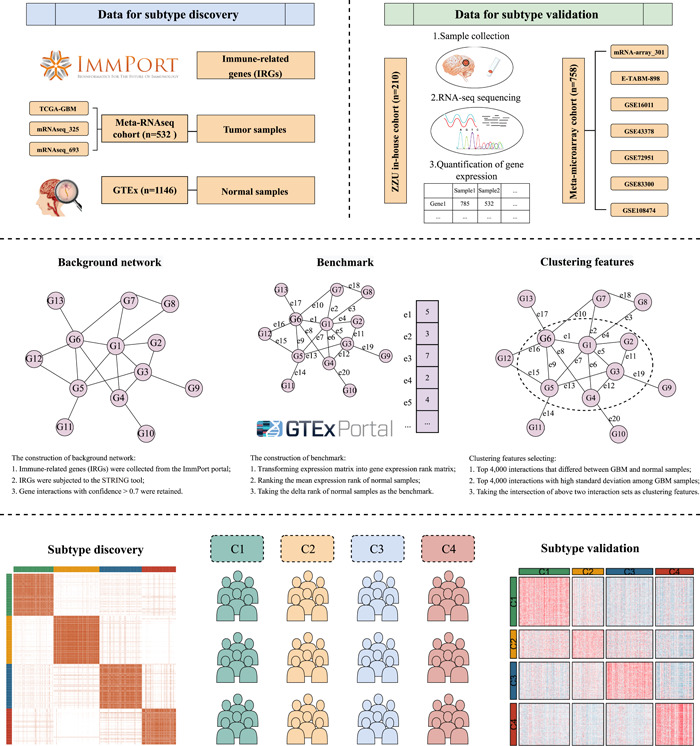
The flowchart of the research progress.

To generate the interaction‐perturbation matrix of immune‐related genes (IRGs), this study employed a previous network‐based pipeline [9]. The protein–protein interactions of IRGs from the STRING tool with confidence >0.7 were regarded as the background network (Figure 1 and Figure S1), which was composed of 1264 nodes and 15,347 edges (Tables S1 and S2). This network was scale‐free with few nodes demonstrating central connectivity (Figure S2A). As previously reported, it is more possible to identify potential subtypes with small fractions from a cohort with a large sample size [5, 7]. Additionally, RNA‐seq data from TCGA and CGGA databases was compatible with the GTEx cohort (RNA‐seq data). Hence, the network‐based pipeline applied the Meta‐RNAseq cohort (batch effects were removed by the *ComBat* algorithm) as GBM sample input and the GTEx cohort as normal sample input. As mentioned above, the expression rank matrix was first obtained by ranking the expression of each gene in each sample and was further converted into the delta rank matrix according to the background network interaction (Figure 1). Previous research has demonstrated that gene interactions in normal tissues were more stable and conservative than in tumor tissues [9]. Thus, we calculated the delta rank vector from the average expression vector of all normal samples as the benchmark of a single sample to generate the interaction perturbation matrix (Figure 1), which could reflect the interaction perturbation of a single sample. As expected, tumor samples displayed stronger perturbations than normal samples (Figure S2B). The mean absolute magnitude of perturbations in GBM samples was 81.4, more than double as high as normal samples. Subsequently, we randomly selected 3000 features and observed that GBM samples displayed a wider variation and higher perturbation relative to normal samples (Figure S2B). The above suggested interaction perturbation could represent the lesion of samples, making it possible to identify the heterogeneity in GBM.

**Figure 2:**
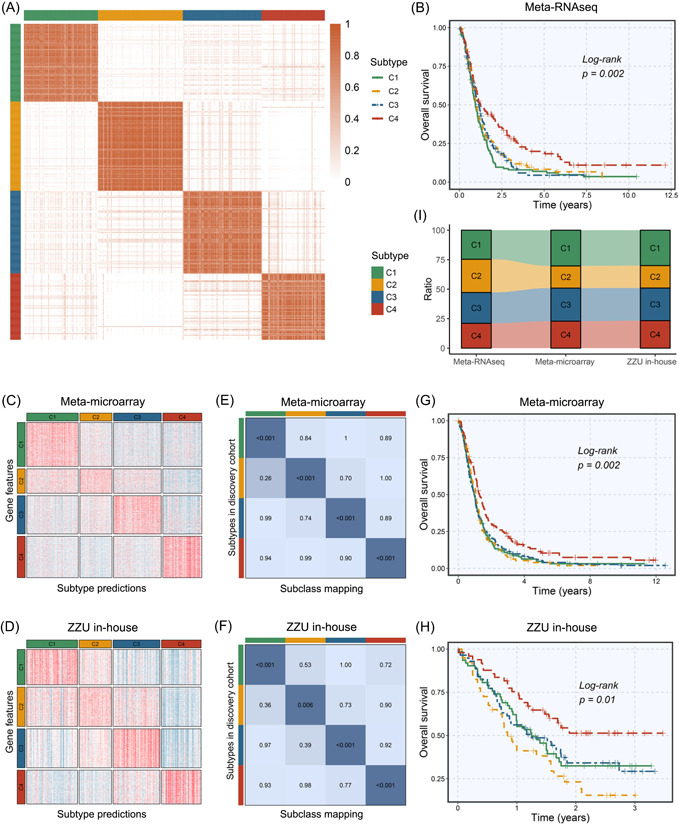
Identification and validation of four immune subtypes based on the gene interaction‐perturbation network. (A) Sample clustering heatmap of the Meta‐RNAseq cohort. (B) Kaplan–Meier of overall survival (OS) with log‐rank test for four subtypes in Meta‐RNAseq cohort. (C, D) The nearest template prediction (NTP) heatmap of Meta‐microarray cohort (C) and Zhengzhou University (ZZU) in‐house cohort (D). (E) The SubMap between Meta‐microarray cohort and the discovery cohort. (F) The SubMap between ZZU in‐house cohort and the discovery cohort. (G, H) Kaplan–Meier of OS with log‐rank test for four subtypes in Meta‐microarray cohort (G) and ZZU in‐house cohort (H). (I) The proportion of C1, C2, C3, and C4 in Meta‐RNAseq cohort, Meta‐microarray cohort, and ZZU in‐house cohort.

Subsequently, we retained 1461 interactions (formed by 606 genes) with predominant perturbation in tumor samples and high heterogeneity to perform consensus clustering [19] (Table S3). Based on the interaction‐perturbation matrix, 532 GBM samples from the Meta‐RNAseq cohort were divided into *k* (2–9) groups via consensus clustering. The consensus matrix, cumulative distribution function (CDF) curve, and delta area plot indicated the optimal number of clusters was 4 (Figure 2A and Figure S2C,D), including C1 (131 patients, 25%), C2 (150 patients, 28%), C3 (139 patients, 26%), and C4 (112 patients, 21%).

Kaplan–Meier survival analysis suggested significant differences in prognosis among four subtypes, in which C4 displayed the most prolonged OS (*p* = 0.002, Figure 2B). As is well established, radiation and chemotherapy are the clinical routines of GBM patients, which have been proven to prolong the median survival of GBM [20]. Here, to evaluate the sensitivity to radiation and chemotherapy of different subtypes, we performed subgroup analysis based on different treatment statuses. For patients without radiotherapy, four subtypes demonstrated no difference in prognosis (*p* = 0.153, Figure S3A). For patients with radiotherapy, there were significant discrepancies in prognosis among four subtypes (*p* = 0.006), and C4 displayed the best prognosis (Figure S3B). Chemotherapy results also demonstrated similar findings (Figure S3C,D). Overall, C4 patients might be more susceptible to routine radiotherapy and chemotherapy than other subtypes, which also explained its favorable prognosis.

### Subtype validation in external and internal cohorts

To validate the reliability and reproducibility of our taxonomy in cross‐platform cohorts, this study retrieved 758 eligible GBM samples from public microarray datasets and performed RNA‐sequencing on 210 GBM samples from our hospital. Initially, 1200 signature genes of four subtypes were recognized by differential expression analysis (Table S4), which were further subjected to the nearest template prediction (NTP) validation framework [21]. Four subtypes identified from the discovery cohort were confidently reassigned in an external Meta‐microarray cohort (Figure 2C) and an internal RNA‐seq cohort (Figure 2D), respectively. Subclass Mapping (SubMap) analysis further confirmed four subtypes from validation datasets shared analogical transcriptional traits with corresponding subtypes from the discovery cohort (Figure 2E,F). Furthermore, C4 exhibited the best prognosis in validation cohorts (Meta‐microarray: *p* = 0.002; Zhengzhou University [ZZU] in‐house: *p* = 0.01), which was concordant with the prior findings (Figure 2G,H). In addition to similar transcriptome and clinical traits, four subtypes also maintained comparable proportions across different cohorts (Figure 2I), further illustrating the reliable performance in cross‐platform cohorts. Overall, validation works from different sequencing techniques and large‐scale data verified the robustness and universality of our taxonomy.

### Biological peculiarities underlying four subtypes

**Figure 3:**
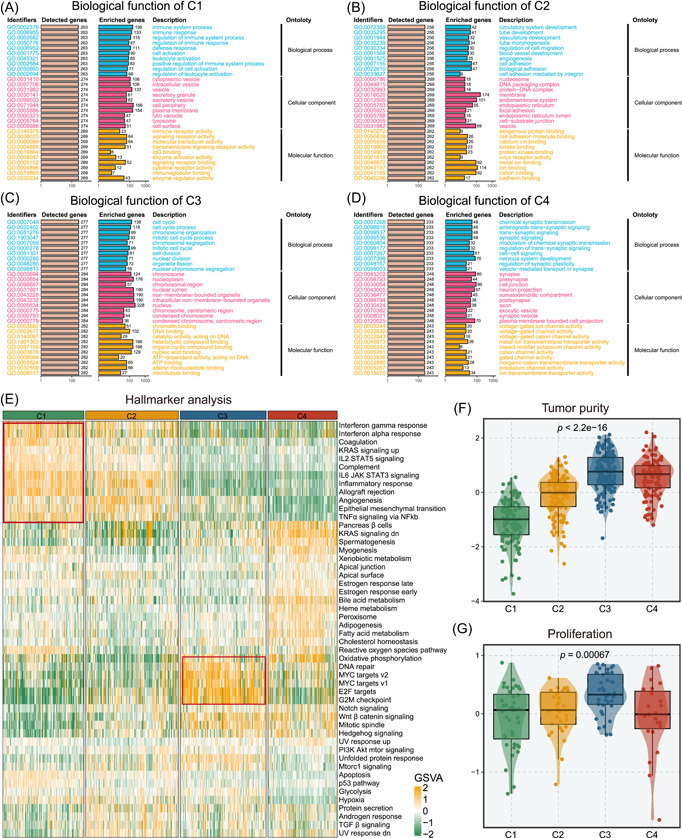
Biological function and immune infiltration of four subtypes. (A–D) The overall survival (OS) analysis of C1 (A), C2 (B), C3 (C), and C4 (D) in Meta‐RNAseq cohort. (E) Gene set variation analysis (GSVA) analysis of “Hallmark” genesets in Meta‐RNAseq cohort, and the enrichment level was represented by *z*‐values. (F, G) The tumor purity (F) (measured by the ESTIMATE algorithm), proliferation score of four subtypes (G) in Meta‐RNAseq cohort.

To further explore the potential biological peculiarities of distinct subtypes, we performed functional enrichment based on overexpression representative analysis (ORA) and gene set variation analysis (GSVA). Specifically, C1 was endowed with high immune activity and low tumor purity (Figure 3A,E,F). C2 displayed tumor invasiveness phenotypes and moderate immune activity (Figure 3B,E). C3 was distinguished by proliferative‐related pathways, such as cell cycle, E2F targets, and G2M checkpoints (Figure 3C,E). Additionally, tumors with high tumor purity were enriched in C3, suggesting the proliferative peculiarity of C3 tumors (Figure 3F,G). C4 was associated with synaptogenesis and signal transmissions containing chemical synaptic transmission, trans‐synaptic signaling, and channel activity (Figure 3D). Moreover, the expression of synapse‐related genes was dramatically overexpressed in C4, demonstrating the synapse formation in C4 (Figure S4). To compare our taxonomy with previous molecular subtypes, we analyzed the corporation of Classical (CL), Mesenchymal (MES), and Proneural (PN) in four subtypes [8]. Specifically, C1 demonstrated superior composition of MES subtype, followed by C2 and C3, while C4 did not encompass MES subtype at all. Conversely, when it came to the CL subtype, C4 exhibited the highest prevalence, followed by C3, C2, and C1 (Figure S5). Collectively, we defined C1 as immune‐infiltrated GBM, C2 as invasive GBM, C3 as proliferative GBM, and C4 as synaptogenesis GBM.

### The difference of metabolism in four subtypes

To explore the metabolism character of four subtypes, we analyzed the enrichment of nine substance‐related pathways via GSVA (Figure S6). Notably, C1 was distinguished by the biodegradation metabolism of exogenous organisms, indicating elevated drug metabolism, which may induce drug resistance. C2 delineated superior level of glucose metabolism, especially glycolysis. The high glycolytic activity could metabolize large amounts of glucose through lactate fermentation, even under aerobic conditions, which can lead to an acidic environment, thereby promoting tumor angiogenesis [22]. Activation of nucleotide metabolism was attributed to C3, which coincided with its high proliferation level. Specifically, C4 was endowed with the enrichment of amino acids and lipid metabolism, which contributed to the synaptic formation and signaling [23, 24]. To sum up, these findings exhibited unique activation of metabolism‐related pathways in four subtypes and offered a higher resolution of our taxonomy.

### Immune landscape and immunotherapeutic potential of four subtypes

**Figure 4:**
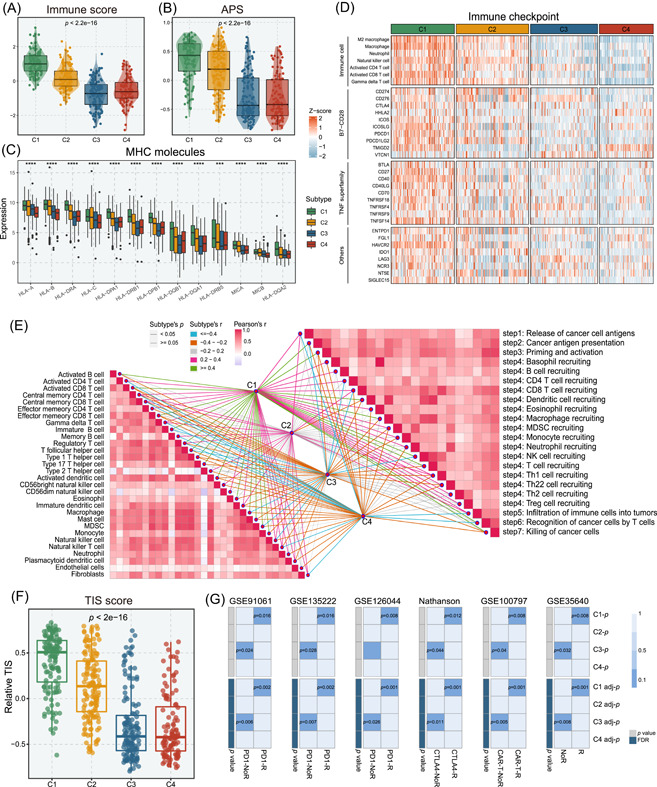
Immune infiltration and immunotherapy prediction of four subtypes. (A) The immune score of four subtypes. (B) The relative antigen processing and presenting machinery score (antigen processing and presenting machinery score [APS]) of four subtypes. (C) The expression of MHC molecular in four subtypes. (D) Heatmap of seven immune cell populations and 27 immune checkpoint molecules for all samples. The relative infiltration abundance of immune cells and the expression of immune checkpoints were represented by *z*‐values. (E) The correlation of four subtypes with the steps in cancer‐immunity cycle and the expression of immune cells. (F) The tumor inflammation signature (TIS) score in four subtypes. (G) SubMap analysis of the four subtypes and six immunotherapeutic cohorts (GSE91061, GSE135222, GSE126044, Nathanson, GSE100797, and GSE35640), all with detailed immunotherapy information. For SubMap analysis, a smaller *p*‐value implied a more similarity of paired expression profiles. ***p < 0.001, ****p < 0.0001.

Four subtypes also demonstrated significant differences in the tumor microenvironment, with C1 conveying higher immune and stromal scores (measured by the ESTIMATE algorithm [25]), which indicated low tumor purity and abundant microenvironment components in C1 (Figure 4A and Figure S7A). To further quantify the immune infiltration across four subfamilies, enrichment levels of 29 immune signatures [26] were profiled through single sample gene set enrichment analysis (ssGSEA) (Figure S7B). C1 showed the highest overall score, followed by C2, and C3 and C4 displayed scarce abundance. The antigen processing and presenting machinery score (APS) [27] and major histocompatibility complex (MHC) molecules were utilized to characterize the antigen processing and presenting capacity in tumors, which delineated higher levels in C1 (Figure 4B,C). Moreover, C1 also processed superior infiltration abundance of immune cells (estimated via ssGSEA), such as CD8+ T cells, CD4+ T cells, and natural killer cells (Figure 4D,E). However, CIBERSORT result demonstrated that the major proportion of immune cell in C1 was M2 macrophages (Figure S7C). We further conducted the correlation between four subtypes and immune cells as well as cancer‐immunity cycle. Clearly, C1 was positively linked to the majority of immune cells and cancer‐immunity cycle [28] (Figure 4E and Figure S8). Intriguingly, C1 exhibited a higher expression of immune checkpoint, costimulatory, and coinhibitory molecules (Figure 4D and Figure S7D,E). Thus, C1 tumors might benefit more from immunotherapy.

To confirm our conjecture, two bioinformatics algorithms, including tumor inflammation signature (TIS) score [29] and SubMap [30], were applied to evaluate the immunotherapeutic efficacy. TIS score was performed to measure the suppressed immune response pre‐existing within tumors [29]. A clinical study of prospective immunotherapy cohorts showed that patients with high TIS scores possessed more benefits from immunotherapy [31]. Here, C1 conveyed the highest TIS score, whereas the lowest score was assigned to C3 (Figure 4F). Furthermore, SubMap analysis was conducted to elucidate the similarity of expression patterns between four subtypes and patients with different responses to immunotherapy [30]. As expected, C1 shared the transcriptional traits with responders from all immunotherapy cohorts (Figure 4G). Conversely, C3 demonstrated analogical expression patterns with nonresponders (Figure 4G). Indeed, tumors with high purity and scarce immune infiltration were significantly enriched in C3, which suggested C3 was featured by the immune‐desert phenotype and insufficient immune reserve for immunotherapy. We also categorized the merged immunotherapy cohort based on the selected feature genes via NTP validation framework and compared the response rate of the four subtypes to immunotherapy. The result was displayed in Figure S9, which was consistent with previous findings. Of note, these immunotherapeutic cohorts were not from GBM patients but could remain reference values for our subtype exploration, as previously reported [32, 33, 34].

The results from multiple bioinformatics approaches described above deciphered that C1 subtype may benefit from immunotherapy, while C3 subtype exhibited a nonresponse to immunotherapy. To further corroborate the findings with experimental evidence, we performed immunohistochemistry (IHC) staining of PD‐L1 across four subtypes. Representative samples were randomly selected in each subtype for PD‐L1 staining and the statistical analysis regarding PD‐L1 expression revealed that C1 demonstrated a notably elevated level of PD‐L1 expression, whereas C3 exhibited a comparatively inferior level (Figure S10A,B).

### Four subtypes conveyed distinct genomic features

**Figure 5:**
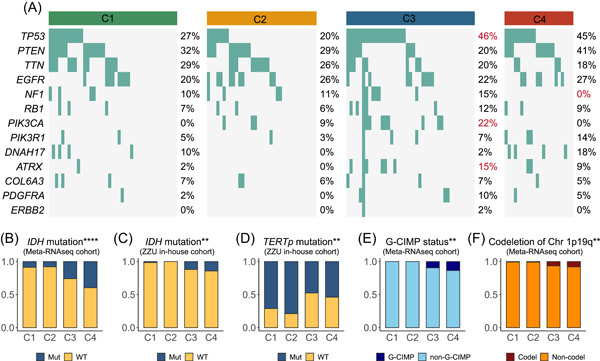
The landscape of genomic features of four subtypes. (A) Waterfall plots of gene mutation in four subtypes. (B) The mutation of isocitrate dehydrogenase (IDH) in Meta‐RNAseq cohort. (C, D) The mutation of IDH (C) and telomerase reverse transcriptase promoter (TERTp) (D) in Zhengzhou University (ZZU) in‐house cohort. (E, F) Comparison of G‐CIMP status (E) and codeletion of chromosome (chr) 1p19q (F) among four subtypes. **p < 0.01, ****p < 0.0001.

GBM was characterized by noteworthy genomic instability and molecular heterogeneity. In this study, *TP53*, *ATRX*, and *PIK3CA* mutations were prevalent in C3 (Figure 5A). *NF1* mutations, which could lead to neurofibromin loss and subsequent elevated RAS activity, were regarded as a biomarker for treatment‐resistant gliomas [35], demonstrating scarce frequency in C4 (Figure 5A). We also compared the proportion of *17q11.2* deletion, which could decrease *NF1* expression (Figure S7F). Of the four subtypes, C2 presented 25% deletion, followed by C1 (21%), C3 (11%), and C4 (5%). Furthermore, the status of O6‐methylguanine methyltransferase (MGMT) promoter was explored in four subtypes, which could improve the sensitivity to chemotherapeutic agents with methylation status [36]. Although the four subtypes did not differ statistically, MGMT promoter methylation increased progressively in the four types, with C4 characterized by the highest MGMT promoter methylation (Figure S11). Moreover, we also compared the mutation frequency of *IDH* and telomerase reverse transcriptase promoter (TERTp) in Meta‐RNAseq and ZZU in‐house cohorts (Figure 5B–D). C4 was endowed with the highest *IDH* mutations, corresponding to the favorable prognosis of C4 (Figure 5B,C). Additionally, *TERTp* mutations, the independent indicator of poor clinical outcome [37], were enriched in C2 (Figure 5D). Furthermore, we observed that G‐CIMP status and codeletion of chromosome (chr) 1p19q status were particularly evident in C4, which were both favorable factors for prognosis, further validating the best clinical outcome of C4 (Figure 5E,F). Overall, these results indicated that four subtypes were featured by distinct genomic traits that may drive different biological peculiarities.

### Potential therapy agents in four subtypes

The above results have deciphered four subtypes with distinct characters, which provided directions to subtype‐based targeted inventions. To better advance clinical treatment, drug prediction was introduced in the present study for the identification of promising therapeutic agents in four subtypes (Figure S12).

Four components targeting *RTK* signaling, axitinib, masitinib, OSI‐930, and pazopanib, were found to have preferential function in C2. Among the *RTK* signaling, *PDGF/RTK* and *VEGF/RTK* are closely related to tumorigenic growth processes, especially in promoting tumor angiogenesis, which was consistent with the invasive peculiarity of C2 [38]. Notably, five agents targeting proliferation were specifically designed for C3, including vorinostat inhibiting chromatin histone acetylation, cisplatin, cytarabine and TMZ inhibiting DNA replication, and MK‐2206 targeting *PI3K* signaling, which also coincide with the proliferative trait of C3. Several types of agents were found to have anticancer activity in C1, such as apoptosis regulation, cell cycle, *JNK* and *p38* signaling, and cytoskeleton. Combined with the finding that C4 was more sensitive to chemotherapy, we hypothesized that C4 may have better responsiveness to multiple chemotherapeutic agents. However, only one agent which was still in clinical development was harbored in C1. This could be attributed to the low purity of the tumor in C1, which hampered the effectiveness of chemotherapy drugs. As previously established, C1 exhibited sensitivity to immunotherapy, which may emerge as the most favorable therapeutic approach for C1. Collectively, we detected subtypes‐specific interventions for four subtypes, which are also concordant with their respective peculiarities.

## DISCUSSION

Cancer cells in GBM generated a proangiogenic and inflamed microenvironment, which recruited immune cells and molecules to infiltrate the tumor mass, leading to intricate immune networks in GBM [39]. Emerging evidence has demonstrated that immune networks consisting of multiple immune components in the tumor microenvironment could characterize the immune phenotypes of tumors and impact the clinical outcomes of GBM patients [17, 18]. Due to the essential role of immune networks and their diverse presence in GBM, this study performed clustering analysis based on IRGs, which may contribute to the understanding of GBM heterogeneity and facilitate individualized treatment.

Gene expression profiles were subject to variation and may exhibit dissimilarities when assessed at distinct time points or under diverse conditions, which results in the instability of subtypes based on expression data. To tackle the snapshot effect of expression profile analysis, we utilized relatively stable gene interaction‐perturbation networks to discover molecular subtypes. In addition, network‐based characteristics have demonstrated high resilience and effectiveness compared to single‐gene features and have been widely accepted for analyzing high‐throughput data [9, 40]. However, most of these approaches only utilize gene sets within a network. Unlike previous pathway‐based approaches, the network‐based approach focused not only on the molecules in gene sets but also on their interactions, reflecting the fact that each molecule does not function independently. Based on immune‐related interaction perturbation networks, four subtypes with different biological characteristics were identified. This taxonomy was proven to be stable and repeatable in cross‐platform datasets, which displayed analogical transcriptome traits, clinical outcomes, and comparable proportions. Furthermore, four subtypes also delineated distinct biological characteristics and molecular interpretability, which might shed light on the clinical stratified management and subtype‐based specific interventions.

C1 was an immune‐infiltrated subtype, with superior immune infiltration, high stromal score, low tumor purity, *NF1* expression deficiency, MES‐like subtype, poor prognosis and potential response to immunotherapy. The high level of APS and enriched infiltration of immune cells tend to activate antitumor immunity in C1 [27]. The positive correlation between C1 and cancer‐immunity cycle, which refers to a series of steps allowed to initiate, proceed and expand for the effective killing of cancer cells in the anticancer immune response [28], further validated the immune‐infiltrated phenotype of C1. The high TIS score in C1 implied its potential to benefit from immunotherapy, which was further verified via SubMap analysis and experimental evidence [30]. However, tumors with elevated expression levels of immune checkpoints and coinhibitory molecules was also enriched in C1, which indicated that C1 may evade immune elimination by overexpressing immunosuppressive agents after stimulating immune activation. Specifically, *NF1* deficiency was found in C1, which was consistent with the high M2 macrophage infiltration. The M2 macrophages subtype in GBM represents tumor supportive macrophages with the ability of promoting tumor development in the neoplastic context, which may partly contribute to the poor prognosis of C1 [41]. The low tumor purity, *NF1* expression deficiency and M2 macrophage infiltration were the characteristics of the MES subtype, which conveyed a worse prognosis [8]. The comparison regarding molecular subtype deciphered the MES‐like feature of C1, coincident with its poor prognosis. Collectively, although C1 was characterized by poor prognosis, the peculiarity of C1 provided insight to screen patients who might benefit from immunotherapy in GBM.

C2 and C3 both demonstrated malignant phenotypes with dismal clinical outcomes. C2 was an invasive subtype, with moderate levels of immune infiltration, plentiful *TERTp* mutations, and sensitivity to *RTK* signaling inhibitors. As a common driver gene of GBM, the mutation of *TERTp* was the independent indicator of poor clinical outcome, leading to the elevated expression level of telomerase and the immortalization of tumor cells [37, 42]. The plentiful *TERTp* mutations in C2 implied the malignant phenotype and were concordant with its dismal prognosis. Align with its invasive feature, four components targeting *RTK* signaling, axitinib, masitinib, OSI‐930 and pazopanib, were found to have preferential function in C2. C3 was a proliferative subtype, with an “immune‐desert” tumor immune microenvironment, high tumor purity, *TP53*, *ATRX* and *PIK3CA* mutations, and sensitivity to *PI3K* signaling inhibitor and DNA replication inhibitors. In line with the proliferative peculiarity, high *PIK3CA* mutations in C3 could activate *PI3K/AKT* signaling and enhance the proliferation of cancer cells, which could explain high sensitivity to *PI3K* signaling inhibitor of C3 [43]. *ATRX* inactivation due to *ATRX* mutations was associated with *TP53* mutations [44], which coincided with the highest mutation frequency of both *ATRX* and *TP53* in C3. In addition, the resistance to immunotherapy of C3 might be due to the scarce infiltration of immune cells and immune molecules, especially PD‐L1, which could not initiate antitumor immunity sufficiently.

C4 was a synaptogenesis GBM, with the best prognosis, “immune‐cold” phenotype, plentiful *IDH* mutations, scarce *NF1* mutations, high chr 1p19q codeletion, MGMT promoter methylation and G‐CIMP, and potential sensitivity to radiotherapy and chemotherapy. Despite being referred to as immunologically “cold,” C4 had the best prognosis of four subtypes. The high *IDH* mutations have proven to be linked to decreased leukocyte chemotaxis and tumor‐associated immune cells, leading to a longer survival time, which was in line with the peculiarity of C4 [45]. The high MGMT promoter methylation, which could lead to inefficient repair of DNA alkylation, was implicated in the sensitivity to chemotherapy of C4 [36]. Analogically, the M2 macrophage was responsible for the resistance to radiotherapy in GBM, and the low infiltration of M2 macrophage in C4 may explain its sensitivity to radiotherapy [8]. Intriguingly, C4 exhibited significant enrichment in synapse formation as well as signal transduction, suggesting C4 tumor cells may have some connection with neurons. Venkatesh et al. showed that tumor cells could communicate electrochemically with neurons via neuron‐glioma synapses in neural circuits and neuronal activity‐induced depolarization of glioma membranes can cause glioma proliferation, whereas electrochemical signaling by pharmacological or genetic blockade can inhibit glioma xenograft growth and prolong survival [46]. Therefore, inhibiting tumor growth by interfering with the regulation of neuronal excitability may be a new potential therapeutic approach to target C4 tumors.

Although our taxonomy was promising, some limitations also existed in our study. First, all samples in this study were retrospective, and further validation of this taxonomy in prospective data should be performed. Second, the biological characteristics of four subtypes are needed to be validated through experiments. Third, these immunotherapeutic cohorts utilized for immunotherapy prediction were not from GBM patients, and clinical GBM cohorts with immunotherapeutic information are required for further verification. Fourth, the background network was constructed via a STRING tool, which only reflected protein–protein interaction. Although the network of interactions between proteins could represent the gene–gene interactions to a great extent, it may interfere with the current result. Finally, due to limitations of public databases, the 2021 WHO classification of CNS tumors was not available in Meta‐RNAseq and Meta‐microarray cohort to define GBM.

## CONCLUSION

We introduced a network‐based pipeline to construct immune‐related interaction perturbation networks and then identified four subtypes with different biological peculiarities and clinical outcomes, improving our understanding of GBM heterogeneity and facilitating clinical stratified management and precise treatment of GBM patients.

## METHODS

### Data sources and processing

#### GBM RNA‐seq data

RNA‐seq data were retrieved from The Cancer Genome Atlas (TCGA, https://portal.gdc.cancer.gov/) and Chinese Glioma Genome Atlas (CGGA, http://www.cgga.org.cn/). A total of 532 samples constituted the discovery cohort consisting of three datasets, including TCGA‐GBM (*n* = 144), mRNAseq_325 (*n* = 139), and mRNAseq_693 (*n* = 249) (Table S5).

#### GBM microarray data

Microarray data were collected from CGGA, ArrayExpress (https://www.ebi.ac.uk/biostudies/arrayexpress/), and Gene Expression Omnibus (GEO, https://www.ncbi.nlm.nih.gov/geo/). A total of 758 samples constituted the external validation cohort consisting of seven datasets, in which mRNA‐array_301 (*n* = 124) was from CGGA, E‐TABM‐898 (*n* = 56) was from ArrayExpress, and GSE16011 (*n* = 165), GSE43378 (*n* = 32), GSE72951 (*n* = 112), GSE83300 (*n* = 50) and GSE108474 (*n* = 219) was from GEO (Table S5).

#### Normal brain RNA‐seq data

The expression profile of 1146 normal brain samples was derived from GTEx Analysis V8, which was downloaded from Genotype‐Tissue Expression (GTEx, https://gtexportal.org/) database, containing RNA sequencing data of normal tissues.

#### ZZU in‐house cohort and RNA sequencing

A total of 210 GBM samples were collected from patients at the First Affiliated Hospital of Zhengzhou University (ZZU) who underwent surgical treatment and written informed consent was acquired from all patients. All included GBM tissues were approved by the Ethics Committee of the First Affiliated Hospital of Zhengzhou University (No. 2019‐KY‐176). The samples met the screening criteria: (1) histopathological identified as GBM; (2) patients did not receive neoadjuvant chemotherapy or radiotherapy; (3) patients with complete clinical information. The qualification and quantification of RNA extracted from GBM tissues were checked as depicted in a previous study [47]. A total amount of 3 µg RNA per sample was leveraged as input material for sequencing libraries preparation, which was generated by accessing NEBNext® UltraTM RNA Library Prep Kit for Illumina® (NEB, USA). After cluster generation using TruSeq PE Cluster Kit v3‐cBot‐HS (Illumina), the library preparations were sequenced on an Illumina Hiseq platform. Clean data with high quality (clean reads) retrieved after quality control of raw data was utilized for downstream analysis. The clean reads were mapped to the reference genome by reference genome and gene model annotation files downloaded from the genome website. The detailed methods were illustrated in Supporting Information. The clinical data of ZZU in‐house cohort was demonstrated in Table S6.

#### Immunotherapy cohorts

Six immunotherapy cohorts were enrolled in our study, GSE91061, GSE135222, GSE126044, GSE100797, GSE35640, and the Nathanson data set [48, 49, 50, 51, 52, 53]. These cohorts were publicly accessible with gene expression data and immunotherapeutic annotations. According to the Response Evaluation Criteria In Solid Tumors (RECIST, v1.1), immunotherapy responders were defined as possessing a complete response (CR) or partial response (PR), and immunotherapy nonresponders were defined as having stable disease (SD) or progressive disease (PD) [54].

#### Multiomics data for TCGA‐GBM

Somatic mutation data were downloaded from the TCGA portal. Somatic variants were detected using the TCGA VarScan2 pipeline.

#### Data processing

(1) The RNA‐seq data from TCGA, CGGA, ZZU in‐house, and GTEx were transformed into transcripts per kilobase million (TPM) and further log_2_ converted. The processed gene expression matrix of ZZU in‐house cohort was demonstrated in Table S7.
(2) Four microarray datasets, including GSE16011, GSE43378, GSE108474, and E‐TABM‐898, were from the same chip platform (Affymetrix Human Genome U133 Plus 2.0 Array). The raw CEL files were normalized and processed through the robust multiarray average (RMA) method implemented in *affy* package. Microarrays from Illumina (GSE72951) and Agilent (mRNA‐array_301, GSE83300) were directly downloaded.
(3) The *ComBat* algorithm implemented in *sva* package was applied to remove the batch effect among different technical platforms [55]. Overall, subtype discovery was performed in the merged RNA‐seq discovery cohort (Meta‐RNAseq cohort) and validated in the merged microarray cohort (Meta‐microarray cohort), our in‐house RNA‐seq cohort (ZZU in‐house cohort), and merged immunotherapy cohort (Figure 1).

### Subtype discovery based on the immune‐related interaction perturbation matrix

To decode the tumor heterogeneity based on the interaction perturbation of immune networks, the immune‐related interaction perturbation matrix was constructed and further analyzed as follows:

(1) Initially, IRGs were extracted from the ImmPort portal (https://www.immport.org/) and were subjected to the STRING tool (https://string-db.org/). Gene interactions with confidence >0.7 were retained for the construction of background networks and further analysis (Figure 1).
(2) As previously reported, we ranked the expression of each gene among all genes for each sample [9]. Thus, the gene expression matrix was transformed into the gene expression rank matrix. According to the background network of gene interactions, the delta rank matrix (rows representing gene interactions; columns representing samples) was generated by subtracting the ranks of two genes in gene pairs connected by gene interactions. For example, we assumed that *R*_A,S_ and *R*_B,S_ respectively represented the expression rank of gene A and gene B in sample S, which were connected by the interaction E. Then the delta rank (*ẟ*_E,S_) was equal to *R*_A,S_ minus *R*_B,S_.
(3) To generate the gene‐interaction perturbation matrix, we ranked the average expression of all normal tissues and calculated the delta rank vector as the benchmark, which was subsequently subtracted by the delta rank of each sample (Figure 1). For example, we assumed $\bar{ẟ}$_E_ represented the rank of interaction E in the benchmark and *ẟ*_E,S_ represented the delta rank of interaction E in sample S, then the perturbation of interaction E in sample S (Δ_E,S_) was equal to *ẟ*_E,S_ minus $\bar{ẟ}$_E_. This study focused on the interaction‐perturbation matrix of tumor tissues to decipher the heterogeneous subtypes in GBM.
(4) Clustering features were selected based on two criteria: being able to distinguish between normal and tumor tissues as well as maintaining significant heterogeneity in GBM. As previously reported, the top 4000 interactions that differed between GBM and normal samples were selected according to the *p*‐value of Wilcox test and the top 4000 interactions with high standard deviation (SD) among GBM samples were also selected [9] (Figure 1). Consensus clustering [19] was performed with the intersections of the above two interaction sets (Figure 1).
(5) In the discovery cohort, Consensus clustering via *ConsensusClusterPlus* package [19] was utilized to decode potential molecular subtypes (Figure 1). The Euclidean was selected as clustering distance and Partitioning Around Medoid (PAM) was chosen as clustering algorithm [56]. To ensure the robustness of the clustering results, 1000 repetitions were performed [56]. The optimal cluster number was determined through the CDF curve.

### Subtype validation

NTP is a flexible class prediction with a confidence assessment based on a single sample [21, 57]. To evaluate the repeatability of the clusters generated from the discovery cohort, GBM patients in the Meta‐microarray cohort and ZZU in‐house cohort were classified using NTP based on the signature genes, which were generated from differentially expressed genes for each subtype. The false discovery rate (FDR) was set to 0.2 [58]. SubMap was an unsupervised method that could estimate the significance of the commonality between two groups with an adjusted *p* < 0.05 indicating the significant similarity [30]. We employed SubMap to estimate the subtype consistency between two validation cohorts and the discovery cohort. The result was demonstrated via *ComplexHeatmap* package [59].

### Statistical analysis

Detailed methods associated with function analysis, immune infiltration assessment, metabolism analysis, cancer‐immunity cycle, TME characterization analysis, drug prediction, IHC staining, and assessment of immunotherapy responses were illustrated in Supporting Information. All data processing, statistical analysis, and plotting were carried out in *R 4.1.3* software. Kaplan–Meier method and log‐rank test were applied to estimate and compare overall survival (OS) among subtypes. Wilcox test or *T*‐test was performed to compare the divergence of continuous variables between the two groups, and Kruskal–Wallis test was utilized to compare data in three or more groups. Chi‐square test or Fisher exact test was conducted for statistics on categorical variables. An FDR test was used to correct *p*‐values. The correlation was determined via Pearson correlation analysis. All statistical *p*‐values were two‐sided. *p* < 0.05 was regarded as statistically significant.

## AUTHOR CONTRIBUTIONS

Zaoqu Liu contributed conceptualization, methodology, and writing—review and editing. Yudi Xu contributed conceptualization, methodology, data curation, investigation, visualization, and writing—original draft. Yuhui Wang contributed conceptualization and writing—review and editing. Siyuan Weng contributed writing—review and editing. Hui Xu contributed writing—review and editing. Yuqing Ren contributed writing—review and editing. Chunguang Guo contributed writing—review and editing. Long Liu contributed conceptualization, methodology, and writing—review and editing. Zhenyu Zhang contributed resources. Xinwei Han contributed conceptualization, writing—review and editing. All authors have read the final manuscript and approved it for publication.

## CONFLICT OF INTEREST STATEMENT

The authors declare no conflict of interest.

## ETHICS STATEMENT

The ethics application (No. 2019‐KY‐176) was approved by the Ethics Committee of the First Affiliated Hospital of Zhengzhou University.

## Supporting information

Supporting information.

Supporting information.

## Data Availability

Public data used in this study are available in TCGA, Chinese Glioma Genome Atlas CGGA, ArrayExpress, GEO, and GTEx. Sequencing data from our hospital are available from the corresponding authors upon request. Supplementary materials (figures, tables, scripts, graphical abstract, slides, videos, Chinese translated version, and update materials) may be found in the online DOI or iMeta Science http://www.imeta.science/.
