# Supplementary material for: Immune‐related interaction perturbation networks unravel biological peculiars and clinical significance of glioblastoma
Source: Imeta. 2023 Jul 16;2(3):e127. doi: 10.1002/imt2.127 (PMC10989959; doi:10.1002/imt2.127)
Supplement: Supplementary file 1 — Supporting information. [file IMT2-2-e127-s001.docx]

**Supporting information to:**

**Immune-related** **interaction** **perturbation networks unravel biological peculiars and clinical significance of g****lioblastoma**

Running Title: Immune-related interaction perturbation networks characterize four glioblastoma subtypes

Zaoqu Liu^1,2,3#^, Yudi Xu^4#^, Yuhui Wang^5#^, Siyuan Weng^1^, Hui Xu^1^, Yuqing Ren^6^, Chunguang Guo^7^, Long Liu^8*^, Zhenyu Zhang^9*^, Xinwei Han^1,2,3*^

^1^Department of Interventional Radiology, The First Affiliated Hospital of Zhengzhou University, Zhengzhou, Henan 450052, China;

^2^Interventional Institute of Zhengzhou University, Zhengzhou, Henan 450052, China;

^3^Interventional Treatment and Clinical Research Center of Henan Province, Zhengzhou, Henan 450052, China;

^4^Department of Neurology, The First Affiliated Hospital of Zhengzhou University, Zhengzhou, Henan 450052, China;

^5^Department of Clinical Laboratory, The Third Affiliated Hospital of Zhengzhou University, Zhengzhou, Henan 450052, China;

^6^Department of Respiratory and Critical Care Medicine, The First Affiliated Hospital of Zhengzhou University, Zhengzhou, Henan 450052, China;

^7^Department of Endovascular Surgery, The First Affiliated Hospital of Zhengzhou University, Zhengzhou, Henan 450052, China;

^8^Department of Hepatobiliary and Pancreatic Surgery, The First Affiliated Hospital of Zhengzhou University, Zhengzhou, Henan 450052, China;

^9^Department of Neurosurgery, The First Affiliated Hospital of Zhengzhou University, Zhengzhou, Henan, 450052, China.

^#^These authors have contributed equally to this work.

^*^Correspondence to:

Department of Interventional Radiology, The First Affiliated Hospital of Zhengzhou University, Henan, 450052, China. Email: fcchanxw@zzu.edu.cn (Xinwei Han).

Department of Neurosurgery, The First Affiliated Hospital of Zhengzhou University, Zhengzhou, Henan, 450052, China. Email: fcczhangzy1@zzu.edu.cn (Zhenyu Zhang).

Department of Hepatobiliary and Pancreatic Surgery, The First Affiliated Hospital of Zhengzhou University, Henan, 450052, China. Email: zzuliulong1997@163.com (Long Liu).

**Supplementary Figures and Supplementary Methods**

- Figure S1
- Figure S2
- Figure S3
- Figure S4
- Figure S5
- Figure S6
- Figure S7
- Figure S8
- Figure S9
- Figure S10
- Figure S11
- Figure S12
- Supplementary Methods


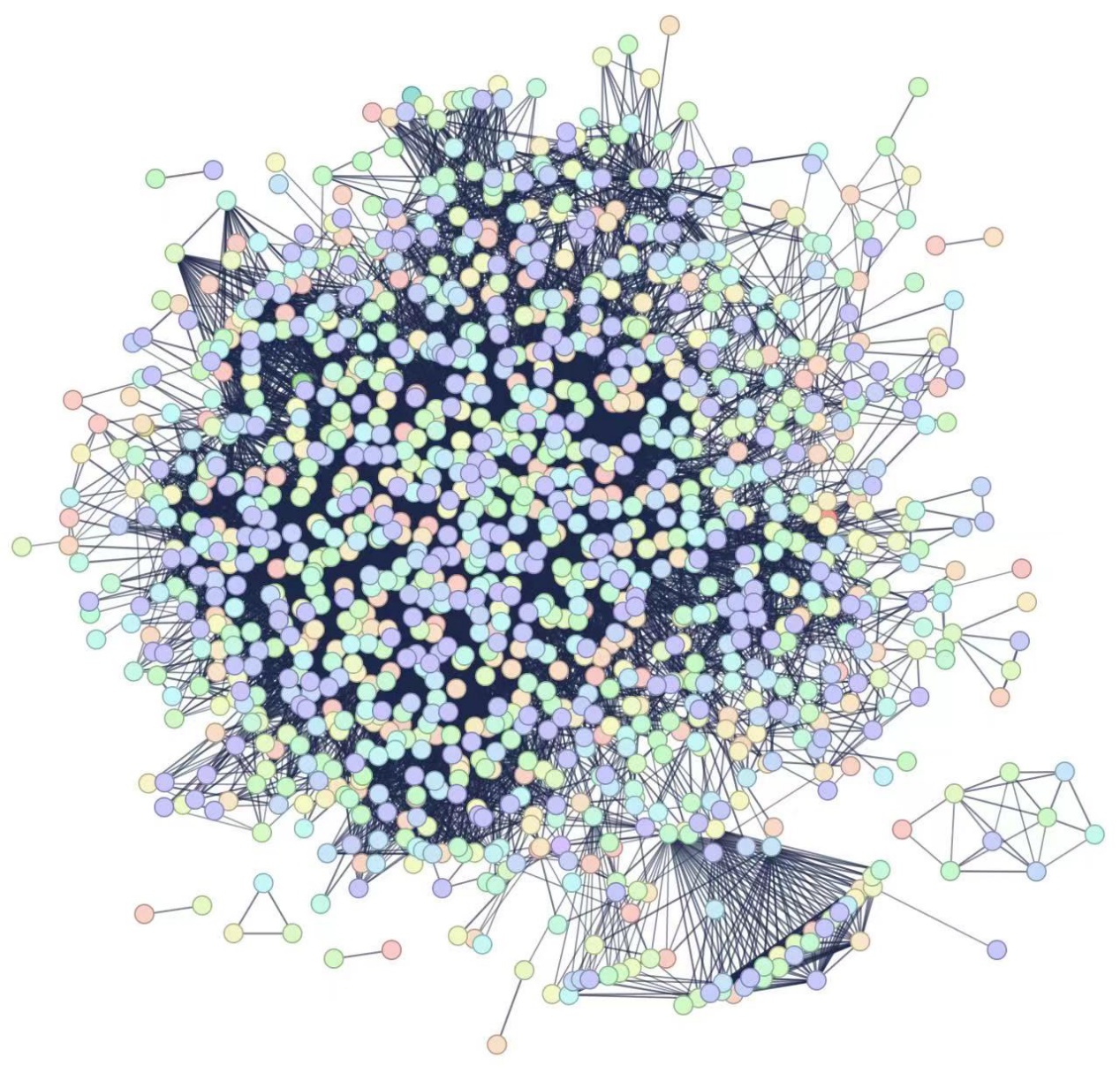


Figure S1. The protein-protein interactions of immune-related genes from the STRING tool with confidence > 0.7.

Figure S2. Construction of gene interaction-perturbation network and identification of potential GBM subtypes. (A) The power distribution of background network. (B**)** The random selected 3000 edge perturbations in tumor and normal samples. **(**C**)** The CDF curves of consensus score from k = 2~10. (D) The delta area under CDF curve from k = 2~10.

Figure S3. The prediction of radiotherapy and chemotherapy sensitivity for glioblastoma subtypes. (A-B) Kaplan-Meier of OS with log-rank test for four subtypes in group of patients without radiotherapy (A) and patients with radiotherapy (B). (C-D) Kaplan-Meier of OS with log-rank test for four subtypes in group of patients without chemotherapy (C) and patients with chemotherapy (D).

Figure S4. The synapse-related gene expression in four subtypes. * *p* < 0.05, ** *p* < 0.01, *** *p* < 0.001, **** *p* < 0.0001. ns, no significance.

Figure S5. The distribution of molecular subtypes (Classical/Mesenchymal/Proneural) in four subtypes.

Figure S6. The difference of metabolism in four subtypes measured by GSVA analysis in Meta-RNAseq cohort. The enrichment level was represented by z-values.

Figure S7. The immune landscape of Meta-RNAseq cohort. (A**)** The stromal score of four subtypes (measured by the ESTIMATE algorithm) in Meta-RNAseq cohort. (B**)** Differential enrichment scores of 29 immune signatures of four subtypes based on ssGSEA. (C-D**)** Box plot of co-stimulatory molecules (C) and co-inhibitory molecules expression (D) in four subtypes. *** *p* < 0.001, **** *p* < 0.0001. ns, no significance.

Figure S8. The correlation between four subtypes and immune cells and cancer-immunity cycles. (A) The correlation between four subtypes and immune cells. (B) The correlation between four subtypes and cancer-immunity cycle. The magnitudes were indicated by the corresponding colors. * *p* < 0.05, ** *p* < 0.01, *** *p* < 0.001, **** *p* < 0.0001. ns, no significance.

Figure S9. The immunotherapy response rate of four subtypes in immunotherapy cohort. *** *p* < 0.001.

Figure S10. The immunohistochemistry (IHC) staining of PD-L1 in the four subtypes of the ZZU cohort. (A) The IHC staining of PD-L1 in the four subtypes of the ZZU cohort. (B) The IHC positive area of four subtypes. ** *p* < 0.01, *** *p* < 0.001.

Figure S11. The status of O6-methylguanine methyltransferase (MGMT) promoter in four subtypes.

Figure S12. The result of drug prediction in four subtypes.

**Supplementary Methods**

**Function analysis and immune infiltration assessment**

Gene Ontology (GO) enrichment analysis was conducted to explore the specific potential biological characteristics in distinct subtypes via *clusterProfiler* package [1]. Gene set variation analysis (GSVA) was a type of gene set enrichment method measuring the enrichment degree of different pathways among different samples via *GSVA* package [2]. To identify the correlated pathways underlying four subtypes, we employed GSVA of the “Hallmark” genesets, which contained comprehensive oncogenic pathways in cancers [3]. He *et al.* introduced 29 immune signatures, representing the overall immune activity of tumors, containing the types, functions and molecular pathways of TIICs [4, 5]. The enrichment levels of those immune gene signatures were quantified by single-sample gene set enrichment analysis (ssGSEA) [2].

**The metabolism analysis**

To distinguish the metabolism characters of four subtypes, we conducted metabolism analysis via GSVA [2]. We enrolled the metabolism-related pathway of nine classes of substances from KEGG, including amino acid, biodegradation metabolism of exogenous organisms, cofactor/vitamin, energy, glycan, glucose, lipid, nucleotide, terpene/polyketones.

**Cancer-Immunity Cycle**

Cancer-Immunity Cycle (CIC) refers to a series of steps allowed to initiate, proceed and expand for the effective killing of cancer cells in the anticancer immune response [6]. Based on the concept that immunity was a dynamic process within tumors, CIC was divided into seven steps [6]. For each step in CIC, ssGSEA was applied to exhibit differences among distinct subtypes.

**TME characterization analysis**

Tumor cell proliferation referred to the process by which tumor cells divide and multiply rapidly, which was one of the hallmarks of cancer and a complex process involving a range of cellular and molecular mechanisms [7]. The score of tumor proliferation was obtained from the research which integrated major immunogenomics methods to characterize the immune tumor microenvironment (TME) across 33 cancers analyzed by TCGA [8].

Tumor purity and immune score respectively represented the proportion of tumor cells and the level of immune cell infiltration in a sample, both calculated via “IOBR” package, which could deconvolute tumor microenvironment on a transcriptomic dataset [9].

**Assessment of immunotherapy responses**

Tumor inflammation signature (TIS), an 18-gene signature, was enriched in patients who responded to PD-1 blockade. TIS score could measure the suppressed immune response pre-existing within tumors to predict immunotherapy efficacy [10]. SubMap was leveraged to infer the commonality of expression profiles between GBM samples and patients with different responses to immunotherapy [11-17].

**RNA samples preparation and sequencing**

**(1) RNA quantification and qualification:** RNA degradation and contamination were monitored on 1% agarose gels. RNA purity was checked using the NanoPhotometer® spectrophotometer (IMPLEN, CA, USA). RNA concentration was measured using Qubit® RNA Assay Kit in Qubit® 2.0 Flurometer (Life Technologies, CA, USA). RNA integrity was assessed using the RNA Nano 6000 Assay Kit of the Bioanalyzer 2100 system (Agilent Technologies, CA, USA).

**(2) Library preparation for Transcriptome sequencing:** A total amount of 3 µg RNA per sample was used as input material for the RNA sample preparations. Sequencing libraries were generated using NEBNext® UltraTM RNA Library Prep Kit for Illumina® (NEB, USA) following manufacturer’s recommendations and index codes were added to attribute sequences to each sample. Briefly, mRNA was purified from total RNA using poly-T oligo-attached magnetic beads. Fragmentation was carried out using divalent cations under elevated temperature in NEBNext First Strand Synthesis Reaction Buffer (5X). First strand cDNA was synthesized using random hexamer primer and M-MuLV Reverse Transcriptase (Rnase H^-^). Second strand cDNA synthesis was subsequently performed using DNA Polymerase I and RNase H. Remaining overhangs were converted into blunt ends via exonuclease/polymerase activities. After adenylation of 3’ ends of DNA fragments, NEBNext Adaptor with hairpin loop structure were ligated to prepare for hybridization. In order to select cDNA fragments of preferentially 150~200 bp in length, the library fragments were purified with AMPure XP system (Beckman Coulter, Beverly, USA). Then 3 µl USER Enzyme (NEB, USA) was used with size-selected, adaptor-ligated cDNA at 37°C for 15 min followed by 5 min at 95 °C before PCR. Then PCR was performed with Phusion High-Fidelity DNA polymerase, Universal PCR primers and Index (X) Primer. At last, PCR products were purified (AMPure XP system) and library quality was assessed on the Agilent Bioanalyzer 2100 system.

**(3) Clustering and sequencing:** The clustering of the index-coded samples was performed on a cBot Cluster Generation System using TruSeq PE Cluster Kit v3-cBot-HS (Illumia). After cluster generation, the library preparations were sequenced on an Illumina Hiseq platform and 125 bp/150 bp paired-end reads were generated.

**(4) Quality control:** Raw data (raw reads) of fastq format were firstly processed through in-house perl scripts. In this step, clean data (clean reads) were obtained by removing reads containing adapter, reads containing ploy-N and low-quality reads from raw data. At the same time, Q20, Q30 and GC content the clean data were calculated. All the downstream analyses were based on the clean data with high quality.

**(5) Reads mapping to the reference genome:** Reference genome and gene model annotation files were downloaded from genome website directly. Index of the reference genome was built using STAR and paired-end clean reads were aligned to the reference genome using STAR (v2.5.1b). STAR used the method of Maximal Mappable Prefix (MMP) which can generate a precise mapping result for junction reads.

**(6) Quantification of gene expression level:** HTSeq v0.6.0 was used to count the reads numbers mapped to each gene. And then FPKM (expected number of Fragments Per Kilobase of transcript sequence per Millions base pairs sequenced) of each gene was calculated based on the length of the gene and reads count mapped to this gene.

**Drug prediction**

GDSC, a pharmacogenomic dataset, stores large-scale drug response and molecular data of human cancer cell lines, enabling accurate prediction of drug response in clinical samples. As previously reported, the model used for predicting drug response was ridge regression model implemented in the *pRRophetic* package [18, 19]. This predictive model was trained on mRNA expression profiles and drug response data of cancer cell lines with a satisfied predictive accuracy were evaluated by default 10-fold cross-validation, thus allowing the estimation of clinical drug response using only patients’ baseline gene expression data. For each subtype, a drug was considered specific for this subtype if its IC50 value for a particular drug was significantly lower (FDR < 0.05) than that of the other two subtypes. We inputted GBM expression profiles into the regression model to estimate the drug response of clinical samples, and ultimately identified promising therapeutic agents for four subtypes.

**Immunohistochemistry (IHC) staining of PD-L1**

Glioma tissue microarrays (NGL1001) were purchased from SUPERBIOTEK (Shanghai Superbiotek Pharmaceutical Technology, Shanghai, China). Clinical data for tumor patients were obtained officially from the company. Immunohistochemistry (IHC) experiments were performed using anti-PD-L1 (1:500; Cat# GB11339A; Servicebio, Wuhan, China). To compare PD-L1 expression statistically, we leveraged IHC Profiler via ImageJ, which could score by staining intensity and staining positivity area.

**REFERENCES**

1. Yu, Guangchuang, Li-Gen Wang, Yanyan Han, and Qing-Yu He. 2012. “clusterProfiler: an R package for comparing biological themes among gene clusters.” *Omics : a journal of integrative biology* 16: 284-287. <https://doi.org/10.1089/omi.2011.0118>

2. Hänzelmann, Sonja, Robert Castelo, and Justin Guinney. 2013. “GSVA: gene set variation analysis for microarray and RNA-seq data.” *BMC bioinformatics* 14: 7. <https://doi.org/10.1186/1471-2105-14-7>

3. Hanahan, Douglas. 2022. “Hallmarks of Cancer: New Dimensions.” *Cancer discovery* 12: 31-46. <https://doi.org/10.1158/2159-8290.Cd-21-1059>

4. He, Yin, Zehang Jiang, Cai Chen, and Xiaosheng Wang. 2018. “Classification of triple-negative breast cancers based on Immunogenomic profiling.” *Journal of experimental & clinical cancer research : CR* 37: 327. <https://doi.org/10.1186/s13046-018-1002-1>

5. Wang, Zihao, Yaning Wang, Tianrui Yang, Hao Xing, Yuekun Wang, Lu Gao, Xiaopeng Guo, Bing Xing, Yu Wang, and Wenbin Ma. 2021. “Machine learning revealed stemness features and a novel stemness-based classification with appealing implications in discriminating the prognosis, immunotherapy and temozolomide responses of 906 glioblastoma patients.” *Briefings in bioinformatics* 22: <https://doi.org/10.1093/bib/bbab032>

6. Chen, Daniel S, and Ira Mellman. 2013. “Oncology meets immunology: the cancer-immunity cycle.” *Immunity* 39: 1-10. <https://doi.org/10.1016/j.immuni.2013.07.012>

7. Whitfield, Michael L, Lacy K George, Gavin D Grant, and Charles M Perou. 2006. “Common markers of proliferation.” *Nature reviews. Cancer* 6: 99-106. <https://doi.org/10.1038/nrc1802>

8. Thorsson, Vésteinn, David L Gibbs, Scott D Brown, Denise Wolf, Dante S Bortone, Tai-Hsien Ou Yang, Eduard Porta-Pardo, et al. 2018. “The Immune Landscape of Cancer.” *Immunity* 48: 812-830.e814. <https://doi.org/10.1016/j.immuni.2018.03.023>

9. Zeng, Dongqiang, Zilan Ye, Rongfang Shen, Guangchuang Yu, Jiani Wu, Yi Xiong, Rui Zhou, et al. 2021. “IOBR: Multi-Omics Immuno-Oncology Biological Research to Decode Tumor Microenvironment and Signatures.” *Frontiers in immunology* 12: 687975. <https://doi.org/10.3389/fimmu.2021.687975>

10. Danaher, Patrick, Sarah Warren, Rongze Lu, Josue Samayoa, Amy Sullivan, Irena Pekker, Brett Wallden, Francesco M Marincola, and Alessandra Cesano. 2018. “Pan-cancer adaptive immune resistance as defined by the Tumor Inflammation Signature (TIS): results from The Cancer Genome Atlas (TCGA).” *Journal for immunotherapy of cancer* 6: 63. <https://doi.org/10.1186/s40425-018-0367-1>

11. Nathanson, Tavi, Arun Ahuja, Alexander Rubinsteyn, Bulent Arman Aksoy, Matthew D Hellmann, Diana Miao, Eliezer Van Allen, et al. 2017. “Somatic Mutations and Neoepitope Homology in Melanomas Treated with CTLA-4 Blockade.” *Cancer immunology research* 5: 84-91. <https://doi.org/10.1158/2326-6066.Cir-16-0019>

12. Riaz, Nadeem, Jonathan J Havel, Vladimir Makarov, Alexis Desrichard, Walter J Urba, Jennifer S Sims, F Stephen Hodi, et al. 2017. “Tumor and Microenvironment Evolution during Immunotherapy with Nivolumab.” *Cell* 171: 934-949.e916. <https://doi.org/10.1016/j.cell.2017.09.028>

13. Kim, Jeong Yeon, Jung Kyoon Choi, and Hyunchul Jung. 2020. “Genome-wide methylation patterns predict clinical benefit of immunotherapy in lung cancer.” *Clinical epigenetics* 12: 119. <https://doi.org/10.1186/s13148-020-00907-4>

14. Siersbæk, Rasmus, Valentina Scabia, Sankari Nagarajan, Igor Chernukhin, Evangelia K Papachristou, Rebecca Broome, Simon J Johnston, et al. 2020. “IL6/STAT3 Signaling Hijacks Estrogen Receptor α Enhancers to Drive Breast Cancer Metastasis.” *Cancer Cell* 38: 412-423.e419. <https://doi.org/10.1016/j.ccell.2020.06.007>

15. Lauss, Martin, Marco Donia, Katja Harbst, Rikke Andersen, Shamik Mitra, Frida Rosengren, Maryem Salim, et al. 2017. “Mutational and putative neoantigen load predict clinical benefit of adoptive T cell therapy in melanoma.” *Nature communications* 8: 1738. <https://doi.org/10.1038/s41467-017-01460-0>

16. Ulloa-Montoya, Fernando, Jamila Louahed, Benjamin Dizier, Olivier Gruselle, Bart Spiessens, Frédéric F Lehmann, Stefan Suciu, et al. 2013. “Predictive gene signature in MAGE-A3 antigen-specific cancer immunotherapy.” *Journal of clinical oncology : official journal of the American Society of Clinical Oncology* 31: 2388-2395. <https://doi.org/10.1200/jco.2012.44.3762>

17. Hoshida, Yujin, Jean-Philippe Brunet, Pablo Tamayo, Todd R Golub, and Jill P Mesirov. 2007. “Subclass mapping: identifying common subtypes in independent disease data sets.” *PloS one* 2: e1195. <https://doi.org/10.1371/journal.pone.0001195>

18. Yang, Chen, Junfei Chen, Yan Li, Xiaowen Huang, Zhicheng Liu, Jun Wang, Hua Jiang, et al. 2021. “Exploring subclass-specific therapeutic agents for hepatocellular carcinoma by informatics-guided drug screen.” *Briefings in bioinformatics* 22: <https://doi.org/10.1093/bib/bbaa295>

19. Geeleher, Paul, Nancy Cox, and R Stephanie Huang. 2014. “pRRophetic: an R package for prediction of clinical chemotherapeutic response from tumor gene expression levels.” *PloS one* 9: e107468. <https://doi.org/10.1371/journal.pone.0107468>
